# ILDGDB: a manually curated database of genomics, transcriptomics, proteomics and drug information for interstitial lung diseases

**DOI:** 10.1186/s12890-020-01350-0

**Published:** 2020-12-11

**Authors:** Yupeng Li, Gangao Wu, Yu Shang, Yue Qi, Xue Wang, Shangwei Ning, Hong Chen

**Affiliations:** 1 Department of Respiratory and Critical Care Medicine, the Second Affiliated Hospital of Harbin Medical University, Harbin, 150081 China; 2 College of Bioinformatics Science and Technology, Harbin Medical University, Harbin, 150081 China; 3 Department of Respiration, Harbin First Hospital, Harbin, 150081 China

**Keywords:** Interstitial lung disease, Gene, ILDGDB, Drug

## Abstract

**Background:**

Interstitial lung diseases (ILDs), a diverse group of diffuse lung diseases, mainly affect the lung parenchyma. The low-throughput ‘omics’ technologies (genomics, transcriptomics, proteomics) and relative drug information have begun to reshaped our understanding of ILDs, whereas, these data are scattered among massive references and are difficult to be fully exploited. Therefore, we manually mined and summarized these data at a database (ILDGDB, http://ildgdb.org/) and will continue to update it in the future.

**Main body:**

The current version of ILDGDB incorporates 2018 entries representing 20 ILDs and over 600 genes obtained from over 3000 articles in four species. Each entry contains detailed information, including species, disease type, detailed description of gene (e.g. official symbol of gene), and the original reference etc. ILDGDB is free, and provides a user-friendly web page. Users can easily search for genes of interest, view their expression pattern and detailed information, manage genes sets and submit novel ILDs-gene association.

**Conclusion:**

The main principle behind ILDGDB’s design is to provide an exploratory platform, with minimum filtering and interpretation, while making the presentation of the data very accessible, which will provide great help for researchers to decipher gene mechanisms and improve the prevention, diagnosis and therapy of ILDs.

## Background

Interstitial lung diseases (ILDs), a diverse group of diffuse lung diseases, mainly affect the lung parenchyma, some of which are characterized by high disabilities and mortality. For instance, idiopathic pulmonary fibrosis (IPF), a common ILD of unknown etiology with repeated acute lung injury, causes gradually progressive lung fibrosis leading to rapidly deteriorated lung function, with mortality of 50% of patients 3–5 years after diagnosis [1–3]. Other ILDs, such as pulmonary sarcoidosis [4, 5], pneumoconiosis [6, 7], connective tissue disease-associated interstitial lung disease (CTD-ILD) [8] and so on also require more healthcare utilization. The pathophysiological mechanism of ILDs is remarkably complex, therefore, it is the primary challenge to discover the precise molecular mechanisms according to genomics, transcriptomics, proteomics etc.

Through the past decades, rapid advances in genetic and genomic technologies have begun to reshape our understanding for ILDs. Studies have uncovered some rare genetic variants such as TERT (telomerase reverse transcriptase) [9, 10], TERC (telomerase RNA component) [10], and some common gene polymorphisms such as MUC5B (mucin 5B, rs35705950) [11] are associated with the development of sporadic IPF or familial interstitial pneumonia (FIP). Changes in gene expression (transcription and protein) levels are also significantly associated with ILDs. For example, TGFB1 is a vital regulator in the progress of ILDs such as IPF, radiation pneumonitis and systemic sclerosis-associated interstitial lung disease (SSc-ILD) et al [12–15]. Some members of chemokine ligands family are also significantly associated with ILDs [16–18]. Currently, several studies have confirmed that nintedanib (an intracellular inhibitor for multiple target genes, including VEGF, FGF, PDGF receptors and so on) is beneficial for patients with IPF [19–21]. Thereby, it is meaningful that new data on potential markers may clarify the pathophysiological mechanism of ILDs, which will promote the development of novel drugs.

At present, with the rapid development of this field, a large number of genes and ILDs data have been accumulated in a short time, whereas, the data are distributed over massive studies, which makes it difficult for researchers to further explore the relationship between ILDs and genes. It is worth noting that some datasets have been developed to explore ILDs-related information. For example, the regulatory model of IPF have been constructed [22] and various single cell RNA-Seq datasets from ILDs were collected at www.ipfcellatlas.com [22–26]. However, there are currently no specialized database focusing on mining experimentally supported gene-ILDs associations among different species. Therefore, ILDGDB, a manually curated database of experimentally supported gene-ILDs associations, was developed to bridge the gap^1^. We expect that ILDGDB will become a useful resource for researchers to explore the relationship between genes and ILDs.

## Construction and content

To ensure a high-quality database, we referred to some high-quality databases such as TBEVHostDB, MGDB, AllerGAtlas 1.0, NSDNA [27–30] etc. Publications were identified through searching the PubMed from January 1, 1900 to April 9, 2018. We screened abstracts of articles obtained from PubMed according to the search strategy (Table 1), then made a list of pertinent articles. Two authors (Y.P.L and Y.S) independently reviewed the full text of the pertinent articles and extracted the data independently, then in duplicate.

**Table 1:** Searched strategy for PubMed

PubMed was searched from 1 January 1900 to 9 April 2018, using the following search strategy	
1. “Lung Diseases, Interstitial”[Mesh] OR Pulmonary Fibrosis* [tiab] OR Idiopathic Interstitial Pneumonias* [tiab] OR pulmonary sarcoidosis* [tiab] OR Interstitial Lung Disease* [tiab] OR Interstitial Pneumonia* [tiab] OR lung fibrosis* [tiab] (*n* = 74,124)	
2. Gene (*n* = 2,439,236)	
3. “1900/01/01”[Date - Publication]: “2018/04/9”[Date - Publication] (*n* = 28,223,179)	
4. 1 AND 2 AND 3 (*n* = 4036)	
5. Review [ptyp] OR meta-analysis [ptyp] OR editorial [ptyp] OR practice guideline [ptyp] OR case reports [ptyp] (*n* = 4,613,012)	
6. 4 NOT 5 (*n* = 3253)	

Genes are obtained from various articles, referring to by different names, share names and symbols, or even gene mentions can be ambiguous, which make gene normalization a challenging task [31]. To overcome the limitation, we made correct association with the Entrez Gene database according to HGNC database (www.genenames.org). Disease normalization is another limitation, therefore, we made correct association with American Thoracic Society/European Respiratory Society (ATS/ERS) classification of idiopathic interstitial pneumonias (IIPs) [32, 33] and the MeSH “Lung Diseases, Interstitial”. Finally, all available data (including regulation of mRNA and protein level, variants, drug information and knockout information) were stored and managed by using MySQL. By using JSP, the web interface was constructed. Java was used for the data processing programs. The web service was developed by using Apache Tomcat. The ILDGDB database is freely available at http://ildgdb.org/.

In the first version of ILDGDB, a total of 2018 entries representing 20 ILDs and over 600 genes in 4 species were manually collected after screening more than 3000 published studies systematically. Each entry contains detailed information, such as disease type, phenotype, detailed description of gene (e.g. official symbol of gene, also known as), species and corresponding literature (title, PubMed ID and publication year) etc. It is worth noting that the data of pure cell lines experiments and high-throughput analysis had not been added into the first version of ILDGDB, however, we plan to add the data into the database in the next version.

## Utility and discussion

**Fig. 1:**
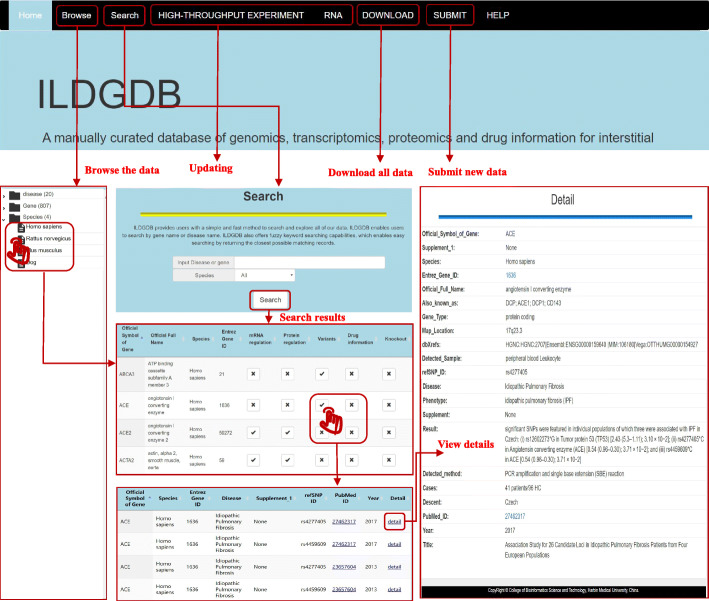
A schematic workflow of ILDGDB

The web interface of ILDGDB is very friendly for users to proceed an easy database query (Fig. 1). Users can browse by official symbol of gene and disease in the ‘Browse’ page. Users can search by symbol of gene and disease in the ‘Search’ page. It is worth noting that fuzzy searching capability is supported by ILDGDB. All possible search results are displayed as tables, and users can click on the ‘Details’ hyperlink to obtain more detailed information in the tables. In the ‘Download’ page, all collected data are free to download. In addition, users can submit novel ILDs-gene associations data in the ‘Submit’ page. Then, the submitted data will be included in the database and serve for the public in the next version after reviewed by our submission review committee. In the ‘Help’ page, a detailed tutorial is provided.

**Fig. 2:**
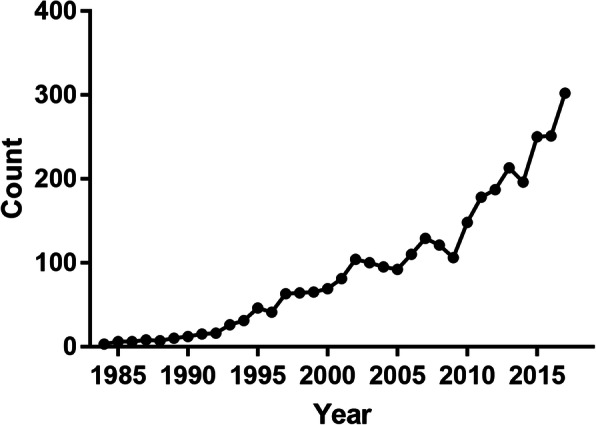
Annual publication counts in PubMed

We counted the number of publications associated with ILDs-related genes each year in PubMed (Fig. 2) and found that the number was rapidly increased, suggesting that more and more researchers and respiratory physicians were trying to decipher the precise molecular mechanisms involved in the development of ILDs. Therefore, the research on genes may be one of the hot topics in the ILDs field in this decade. However, gene-ILDs associations data are dispersed in various published articles. Therefore, a high-quality database with comprehensive ILDs-associated genes data is critical to fully understand the ILDs processes. Some related databases [22–26, 29] had been constructed to enhance our understanding for ILDs, whereas, they only documented little related data and didn’t provide a comprehensive resource on diverse gene-ILDs associations among various species. For example, AllerGAtlas 1.0, a manually curated database for human allergy-related genes, only documents several ILDs and little related gene data, for instance, only 15 genes were included in IPF [29]. Therefore, we developed an ILDs-specific database named as ILDGDB with comprehensive data among four species.

In addition to collecting more gene-ILD associations, ILDGDB has several advantages compared with previous studies. First, ILDGDB includes detailed genes information (official symbol of gene, Entrez Gene ID, official full name, also known as, gene type, map location and dbXrefs) and articles information (as described in database content). Second, ILDGDB includes data for four species and provides a user-friendly web interface for users to retrieve and download all available data. Third, data on gene-associated variants, targeted drug and knockout information were also added to the ILDGDB. Therefore, ILDGDB is a specialized database with comprehensive resource on gene-ILDs associations.

**Fig. 3:**
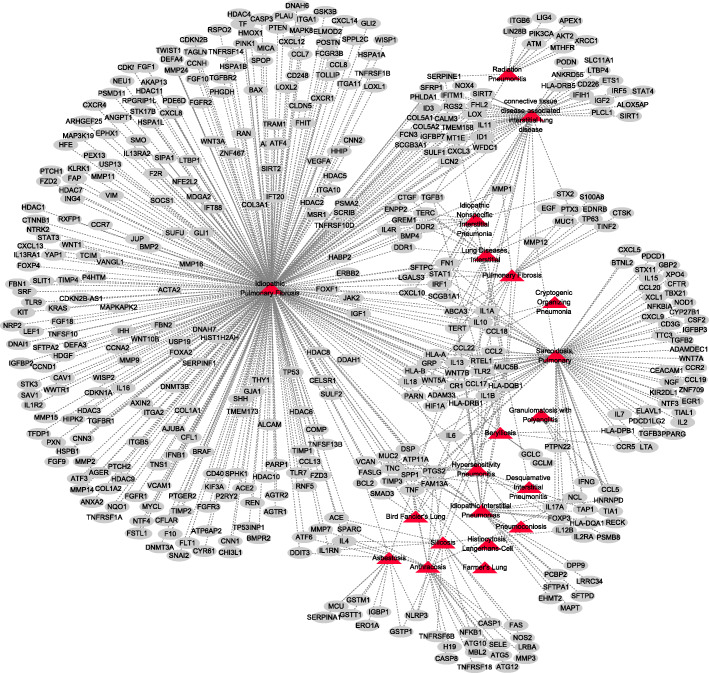
The human gene-ILDs bipartite network. The network is composed of 20 ILDs, 450 human genes and 616 gene-ILDs associations. Triangles and ellipse represent ILDs and genes, respectively. The lines between genes and diseases correspond to experimentally supported associations

ILDGDB includes more than half of human gene-associated data, therefore, we constructed a human gene-ILDs bipartite network according to Cytoscape (a software platform for visualizing complex networks, version 3.7.1) [34], where nodes represent genes or ILDs and the lines represent experimentally supported associations between genes and ILDs (Fig. 3). From the network, we found that IPF is the highest connected disease node with 330 genes associations, which indicates that IPF has received wide attention in gene-related study and also has a complex molecular mechanism regulated by gene. In addition, the highest connected gene node is TNF that is associated with 10 ILDs, which suggests that TNF might be widely associated with ILDs.

At present, we are collecting related data and planning to update ILDGDB. The next version will include these contents as follow: the update of newly validated gene-ILDs associations; integration of high-throughput datasets; integration of RNA data; integration of gene/RNA expression data of pure cell lines; integration of gene/RNA expression data of approved therapies (pirfenidone and nintedanib) or therapies under investigation in Phase III trials (pamrevlumab, GLPG-1690) and so on.

## Conclusions

In conclusion, researchers and respiratory physicians have been trying to decipher the complex regulatory mechanism of ILDs for years. Currently, more and more studies have clearly clarified the gene’s role in ILDs and the related mechanisms. With the support of experimental data, ILDGDB provides not only a comprehensive ILDs-specialized database but also a more global perspective on genes functions in ILDs. In the future, we will continue to update the database every 2-3 years. Furthermore, we plan to integrate more sources and information such as RNA data and provide a gene-ILDs association prediction tool. We believe that ILDGDB will provide great help for researchers to decipher gene mechanisms and improve the diagnosis and therapy of ILDs as a valuable resource.

## Data Availability

The datasets generated and/or analysed during the current study are available in the ILDGDB repository, http://ildgdb.org/.

## Abbreviations

ILDs: Interstitial lung diseases
IPF: Idiopathic pulmonary fibrosis
CTDs: Connective tissue diseases
CTD-ILD: Connective tissue disease-associated interstitial lung disease
FIP: Familial interstitial pneumonia
TERT: Telomerase reverse transcriptase
TERC: Telomerase RNA component
MUC5B: Mucin 5B
IIPs: Idiopathic interstitial pneumonias
SSc-ILD: Systemic sclerosis-associated interstitial lung disease

## Footnotes

1 Database URL: http://ildgdb.org/
